# Case report: Ultrasonographic findings of retroperitoneum and abdominal wall metastases of renal cell carcinoma with FH gene deletion

**DOI:** 10.3389/fonc.2022.896477

**Published:** 2022-10-18

**Authors:** Xuhui Zhang, Yue Zhang, Yongzhong Li, Pengfei Shen, Zhenghua Liu, Hao Zeng, Mengni Zhang, Ni Chen, Jin Yao, Rui Huang, Diming Cai

**Affiliations:** ^1^ Department of Ultrasound Medicine, West China Hospital, Sichuan University, Chengdu, China; ^2^ Department of Urology, West China Hospital, Sichuan University, Chengdu, China; ^3^ Department of Pathology, West China Hospital, Sichuan University, Chengdu, China; ^4^ Department of Radiology, West China Hospital, Sichuan University, Chengdu, China; ^5^ Department of Nuclear Medicine, West China Hospital, Sichuan University, Chengdu, China

**Keywords:** case report, ultrasound, fumarate hydratase-deficient renal cell carcinoma, metastasis, imaging

## Abstract

Renal cell carcinoma with FH gene deletion is a rare subtype of renal cell carcinoma. There had been few reports about ultrasonographic imaging of metastasis of renal cell carcinoma with FH gene deletion. This case reported one of the features of metastasis of renal cell carcinoma with FH gene deletion of a male patient 7 months after undergoing radical nephrectomy. He was diagnosed with a renal malignant tumor before the operation and confirmed to be primary FH gene-deficient renal cell carcinoma after undergoing radical nephrectomy in another hospital. Reexamination 7 months after the operation indicated that multiple metastases all over the body were found; therefore, he came to our hospital for further diagnosis and therapy. The tumors have metastasized in the lungs, bones, and lymph nodes adjacent to the left reproductive vessels and external iliac vessels, retroperitoneum, and abdominal wall so far as confirmed by PET/CT or MRI. Ultrasonographic findings of masses in the retroperitoneum and abdominal wall are fully discussed, which have been confirmed by biopsy and diagnosed as renal cell carcinoma with FH gene deletion by pathology.

## Introduction

Renal cell carcinoma with FH gene deletion is a rare subtype of renal cell carcinoma (RCC). These tumors are usually metastatic at presentation and typically solitary and unilateral, unlike other familial RCC syndromes that are characterized by bilateral multifocal tumors (1). Many patients who presented with local or distant metastasis succumbed to the disease <5 years from the initial diagnosis (2). There had been few reports about ultrasonographic imaging of metastasis of renal cell carcinoma with FH gene deletion. This case reported one of the features of metastasis of renal cell carcinoma with FH gene deletion of a male patient 7 months after undergoing radical nephrectomy.

## Case description

A 36-year-old man presented with a left lumbar mass of 1-month duration without obvious inducement 7 months after undergoing radical nephrectomy. The present study was performed in accordance with the Declaration of Helsinki and was approved by the Ethics Committee of our hospital. In another hospital, the tumor in the left kidney was diagnosed as renal malignancy preoperatively, which was proved to be primary FH gene-deficient renal cell carcinoma after radical nephrectomy. Seven months after the operation, the patient came to our hospital because of multiple metastases in a large number of organs diagnosed by MRI, which were hard, mobilized poorly, and progressively enlarged. The tumor in the abdominal wall near the left waist was resected in our hospital, and targeted therapy and immunotherapy were performed postoperatively. The patient’s parents are alive, his brothers or sisters are in good health, and there is no family or genetic history.

Physical examination showed that the shape of the abdomen was normal and that there was no tenderness or rebound pain. During palpation of the waist of the left kidney area, a hard and poorly mobilized lump that was as big as an egg was found.

Clinical biochemical test (normal values are given in parentheses) showed that creatinine (68–108) was 151 μmol/L, estimated glomerular filtration rate (56–122) was 50.46 ml/min/1.73 m^2^, cystatin-C (0.51–1.09) was 1.64 mg/L, uric acid (240–490) was 572 μmol/L, triglyceride (0.29–1.83) was 4.42 mmol/L, anion gap (12.0–20.0) was 22.2 mmol/L, and calcium (2.11–2.52) was 2.54 mmol/L.

### The features of ultrasound

**Figure 1:**
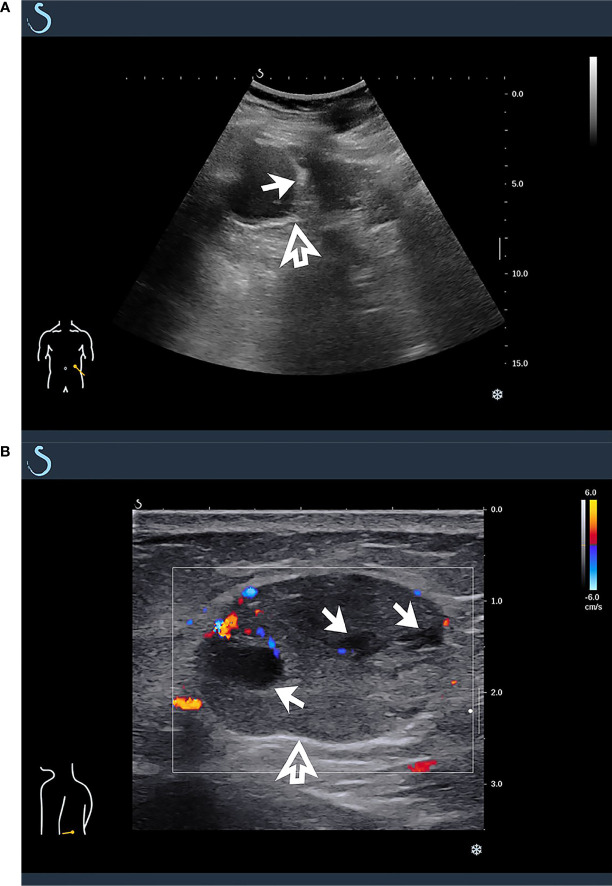
Ultrasonographic findings of masses in retroperitoneum and abdominal wall. **(A)** A multicystic mass was found in retroperitoneum of left renal region by grayscale using low-frequency transducer (hollow arrow), which featured multiple septa inside the cystic component (solid arrow). **(B)** By high-frequency probe, a hypoechoic mass was detected in abdominal wall near the left waist, and there were punctiform and linear blood flow signals in color Doppler (hollow arrow), which featured patchy anechoic areas inside the mass (solid arrow).

**Figure 2:**
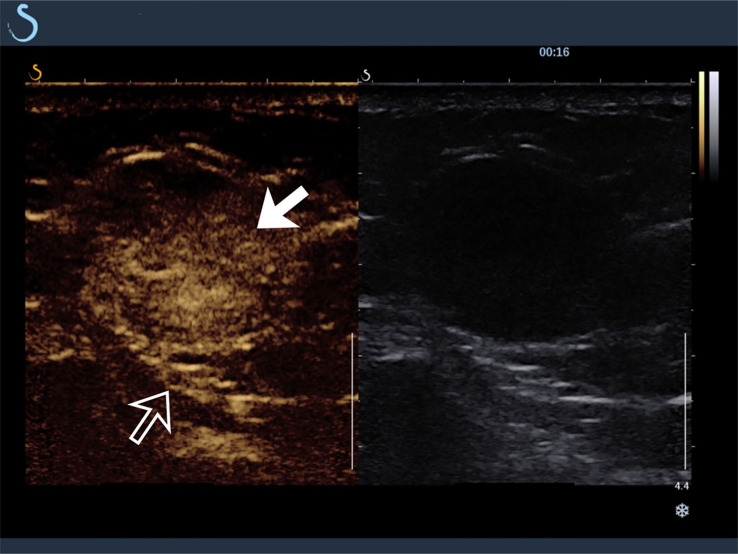
Contrast-enhanced ultrasound findings of mass in abdominal wall near the left waist (hollow arrow). The hypoechoic mass showed rapid hyperenhancement in arterial phase (solid arrow).

The tumors in the retroperitoneum of the left renal region and the abdominal wall near the left waist were probed by ultrasonography. Ultrasonographic images (Aixplorer US system; SuperSonic Imagine, Aix-en-Provence, France) showed a septal cystic mass of about 8.9 × 4.6 × 5.3 cm with an ill−defined margin, relatively regular shape, and heterogeneous echo in the retroperitoneum of the left renal region by grayscale using a low-frequency transducer (SL 6-1 multifrequency convex transducer) (**Figure 1A**). The septum featured an uneven thickness, with 0.6 cm as the thickest. There were punctiform and linear color Doppler signals in the mass. The ultrasound images also showed a hypoechoic mass of about 2.4 × 1.9 × 3.0 cm with a relatively circumscribed margin, regular shape, and heterogeneous echo in the abdominal wall near the left waist, and patchy anechoic areas could be seen inside the mass by grayscale using a high-frequency probe (SL 10-2 multifrequency linear probe). There were punctiform and linear blood flow signals in the mass in both color Doppler (**Figure 1B**) and power Doppler. Contrast-enhanced ultrasound showed that the septum of the cystic mass in the retroperitoneum displayed rapid hyperenhancement in the arterial phase, equal enhancement in the venous phase, and equal enhancement in the delayed phase after intravenous injection of ultrasound contrast agent SonoVue (Bracco SpA, Milan, Italy). The part of the cyst was not enhanced in three phases. Contrast-enhanced ultrasound also showed that the mass in the abdominal wall displayed rapid hyperenhancement in the arterial phase (**Figure 2**), slightly low enhancement in the venous phase, and low enhancement in the delayed phase, and anechoic areas showed non-enhancement in three phases. Ultrasonography suggested that there was a septal cystic mass in the retroperitoneum of the left renal region, and combined with the features of contrast-enhanced ultrasound, it was considered Bosniak grade IV, which could be ascribed to tumor recurrence possibly; the mass in the abdominal wall near the left waist was considered tumor metastasis possibly combined with the features of contrast-enhanced ultrasound (3, 4).

**Figure 3:**
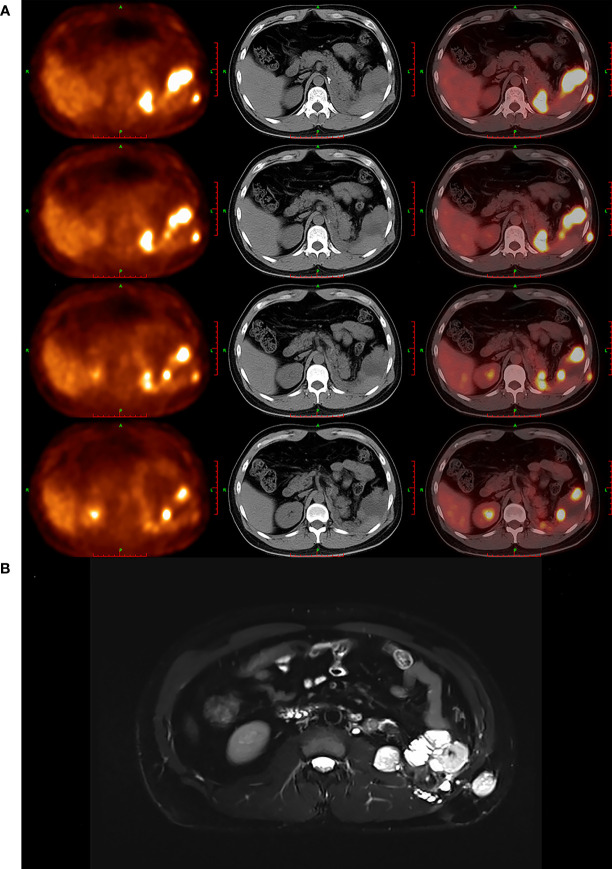
PET/CT and MRI findings of masses. **(A)** PET/CT images showed that multiple soft-tissue-density nodules and masses were found in left renal and adrenal region, perisplenic region, left retroperitoneum, left intraabdominal fascia, and left abdominal wall, which featured high uptake of ^18^F-FDG abnormally. **(B)** MRI images showed that multiple nodules and masses were found in left renal and adrenal regions, perisplenic region, left retroperitoneum, left vertebral fascia, subcutaneous and muscular layer of left posterior abdominal wall, anterior part of left psoas major muscle, and left iliac fossa, which suggested that the tumor has recurred and metastasized likely.

In addition to ultrasonographic findings, other findings of imaging modalities, including PET/CT and MRI, are also provided in **Figure 3**.

The intraoperative discovery showed that the mass was removed completely after dissociating the mass in the subcutaneous connective tissue and adipose tissue layer carefully. There was a capsule in the resected tumor, which has soft tissue and hemorrhagic necrotic foci inside.

**Figure 4:**
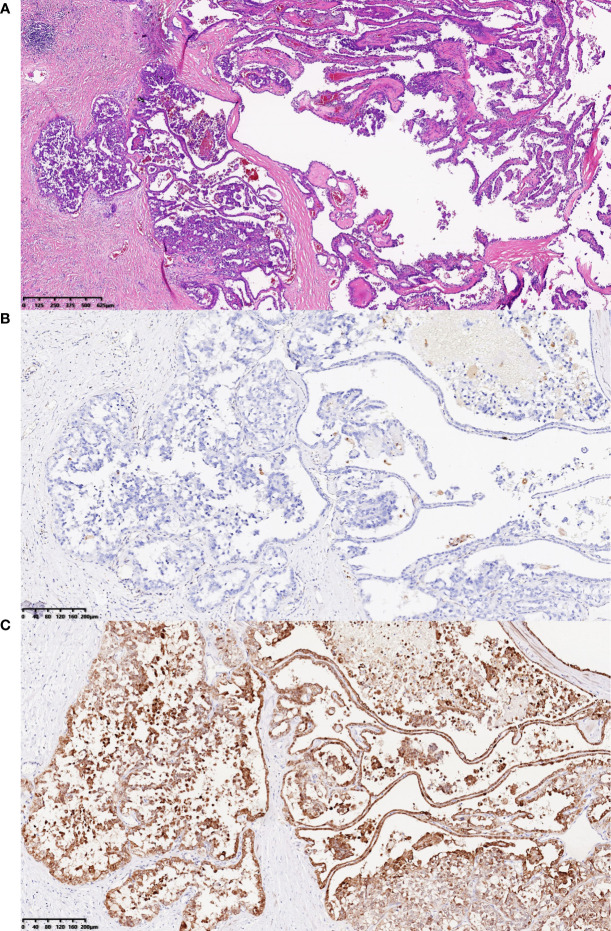
H&E staining results and immunohistochemistry staining results for FH gene and 2SC. **(A)** H&E staining results showed that the tumor cells in abdominal wall near the left waist, the majority of which were arranged into papillary shape, featured abundant and eosinophilic cytoplasm, round or oval enlarged nuclei with marked eosinophilic nucleoli, and perinucleolar halo (hematoxylin and eosin staining; magnification, ×40). **(B, C)** The tumor cells in abdominal wall near the left waist were visualized by immunohistochemistry staining for FH gene and 2SC, which revealed that the expression of FH protein was lost and 2SC was cytoplasmic positive (magnification, both ×100).

Pathological examination showed that the size of the grayish-yellow and grayish-brown nodular mass was 3.5 × 2.5 × 2.5 cm, which had a complete and smooth capsule. The incanus and grayish-brown section plane were solid with moderate texture and hardness. Immunohistochemistry showed that PAX8 was positive, 2SC was positive, AKR1B10 was positive, AMACR was positive, CA9 was negative, CK7 was negative, TTF-1 was negative, and the targeted area was weakly positive with loss of FH gene expression, while the control is positive. The pathological findings were consistent with the metastasis of FH gene-deficient renal cell carcinoma, accounting for about 80% of the tumor (**Figure 4**).

## Discussion

Fumarate hydratase-deficient renal cell carcinoma (FH-d RCC) is a rare subtype of RCC associated with fumarate hydratase (FH) gene and hereditary leiomyomatosis and renal cell carcinoma (HLRCC) syndrome. HLRCC syndrome is a rare autosomal dominant disorder related to germline mutations of FH gene, which increases the risk of developing skin leiomyoma, uterine leiomyoma, and renal cell carcinoma (5, 6). In the latest 2016 edition of WHO renal tumor classification, HLRCC-associated RCC is listed as a new independent subtype of RCC (7).

In humans, the FH protein is encoded by FH gene, which is located on the q-arm of chromosome 1 (8). FH gene participates in the mitochondrial tricarboxylic acid cycle (9). In tumor tissues, heterozygous deletion of FH gene locus often results in complete loss of FH function. The most immediate effect is the accumulation of intracellular fumarate, which at high concentrations directly alters various cellular signaling pathways (8). Pyruvate has difficulty completing the tricarboxylic acid cycle in the cell, and the process of oxidative phosphorylation and energy supply inside the mitochondria is correspondingly difficult to unfold, and the energy supply can only rely on anaerobic glycolysis, which in turn leads to pseudo-hypoxia. There is an inextricable relationship between tumor development and pseudo-hypoxia (10).

At present, most studies focus on clinicopathology, molecular analysis, treatments, and so on of FH-d RCC. Yang et al. (11) investigated the multidetector computed tomography (MDCT) features of FH-d RCC, and Nikolovski et al. (12) evaluated the imaging features of FH-d RCC on CT, MRI, and FDG PET. However, the clinical imaging findings of metastasis of FH-d RCC, such as imaging of ultrasound, are rarely involved in reports.

FH-d RCC is different from ordinary familial RCC. It is highly aggressive, prone to local progression and metastasizing, and has a poor prognosis (13, 14). Studies by Muller et al. and Pan et al. showed that the median survival for metastatic FH-d RCC was about 18 months (15, 16). In this case, the patient underwent radical nephrectomy because of FH-d RCC, and tumors have been recurring and metastasizing in multiple organs 7 months after the operation. By using axitinib for the targeted therapy (200 mg ivggt q21d) and sintilimab (5mg bid) for the immunotherapy, the basic conditions of the patient improved gradually. To date, the patient has undergone 14 months (a total of 18 cycles of sintilimab) of treatment with this regimen, and the patient was very satisfied with our treatment plans.

The imaging features of the tumor or metastasis are unclear as a result of scarcity. The metastases of recurring lesions were reported, which displayed features by ultrasonography. In this case, by grayscale ultrasound, the mass features a septum in the cystic component or patchy anechoic areas in the hypoechoic mass. Relatively circumscribed margin, relatively regular shape, and heterogeneous echo in the mass could provide some clues for the diagnosis. In color Doppler and power Doppler, tiny punctiform and linear blood flow signals may indicate some characteristics of blood supply in the tumor. In contrast-enhanced ultrasound, the features of tumor perfusion, in this case, are distinctive. Hyperenhancement of the whole lesion was observed in the arterial phase, which contradicts ultrasonographic findings in color Doppler and power Doppler. Meanwhile, the tumor detected heterogeneous enhancement with fireworks-like enhancement, which showed punctiform hyperenhancement firstly from the center of the mass and then spread outward like fireworks in spite of patchy anechoic areas, which should present non-enhancement normally.

FH-d RCC is an infrequent renal malignant tumor, which is difficult to diagnose before the operation, and multimodal ultrasonography for the disease is even rarer. In this case, multimodal ultrasonographic features of metastatic lesions were described in detail, including grayscale, color Doppler, power Doppler, and contrast-enhanced ultrasound. Regrettably, there is no multimodal ultrasonography for the primary foci of FH-d RCC in this case report, and more cases are needed to obtain a more comprehensive understanding of the disease.

## Data availability statement

The original contributions presented in the study are included in the article/**Supplementary Material**. Further inquiries can be directed to the corresponding author.

## Ethics statement

The studies involving human participants were reviewed and approved by West China Hospital, Sichuan University. The patients/participants provided their written informed consent to participate in this study.

## Author contributions

XZ, YZ, and YL contributed the part of ultrasound. PS, ZL, and HZ provided the clinical case. MZ, NC contributed the part of pathology. JY contributed the part of MRI. RH contributed the part of PET/CT. DC acted as corresponding author. All authors contributed to the article and approved the submitted version.

## Conflict of interest

The authors declare that the research was conducted in the absence of any commercial or financial relationships that could be construed as a potential conflict of interest.

## Publisher’s note

All claims expressed in this article are solely those of the authors and do not necessarily represent those of their affiliated organizations, or those of the publisher, the editors and the reviewers. Any product that may be evaluated in this article, or claim that may be made by its manufacturer, is not guaranteed or endorsed by the publisher.
