# Supplementary figures and images for: Case report: Ultrasonographic findings of retroperitoneum and abdominal wall metastases of renal cell carcinoma with FH gene deletion
Source: Front Oncol. 2022 Oct 18;12:896477. doi: 10.3389/fonc.2022.896477 (PMC9623255; doi:10.3389/fonc.2022.896477)

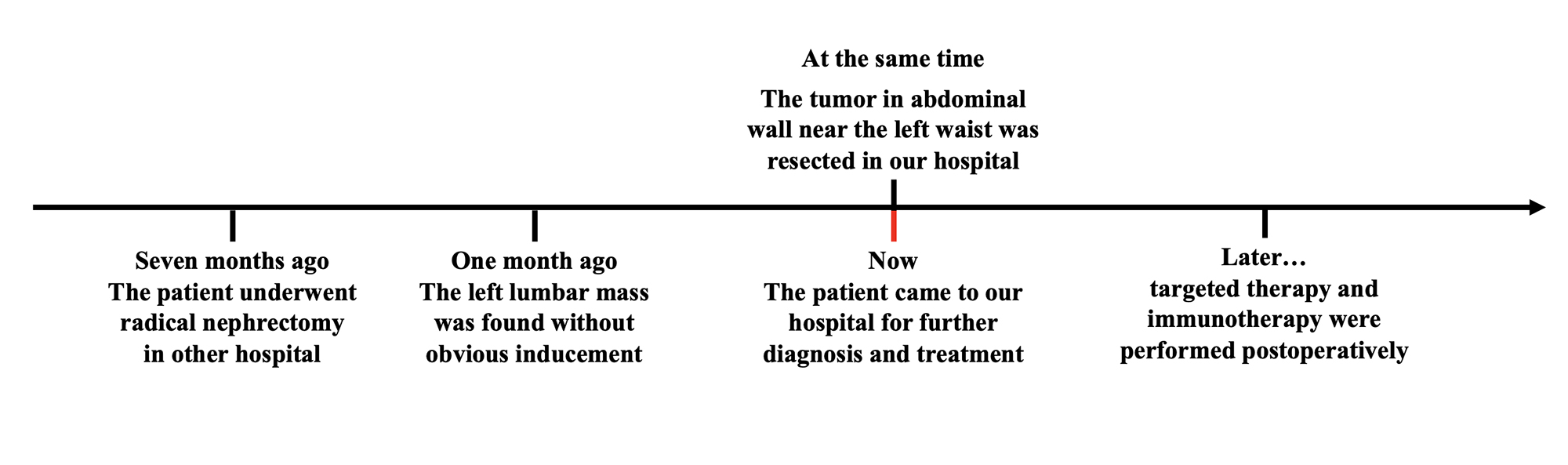

Supplement: Supplementary file 1 [file Image_1.jpg]
